# Early signs of long-term pain: prospective network profiles from late adolescence and lifelong follow-up

**DOI:** 10.1038/s44184-025-00122-0

**Published:** 2025-02-13

**Authors:** William Hedley Thompson, Emelie Thern, Filip Gedin, Anna Andreasson, Karin B. Jensen, Maria Lalouni

**Affiliations:** 1 Department of Applied Information Technology, University of Gothenburg, Gothenburg, Sweden; 2 Department of Clinical Neuroscience, Karolinska Institute, Stockholm, Sweden; 3 Department of Psychology, Stockholm University, Stockholm, Sweden; 4 Institute of Environmental Medicine, Unit of Occupational Medicine, Karolinska Institute, Stockholm, Sweden

**Keywords:** Risk factors, Pain, Chronic pain

## Abstract

This study applies network theory to registry data to identify prospective differences between individuals who develop long-term pain later in life and those who do not. The research is based on assessments of biological, psychological, and social variables in late adolescence during military conscription in Sweden. The analysis reveals significant differences in the network profiles of adolescent men who later developed long-term pain. These differences are reflected in several network-based outputs, including global, nodal, and edge levels, revealing a consistent picture of the pain-associated network profile. This profile demonstrates how those vulnerable to long-term pain have a specific configuration of variables that skew away from the rest of the population, mainly relating to psychosocial aspects.

## Introduction

Long-term pain and mental health problems represent by far the largest share of disability and injury burden today^1^. Long-term pain causes disability in patients of all ages, leading to substantial suffering, an enormous economic burden, and loss of productivity in working-age groups^2^. At any given moment, over 20% of the population experiences long-term pain^3^. However, treatment options are limited because traditional analgesics are typically ineffective for long-term pain^4^. Therefore, to avoid chronicity, identifying those with high risk is essential to provide preventive care and decrease pain incidence, and is a public health priority in the United States^5^. Despite this need, there are currently no definitive demographics or clinical tests to predict who will develop long-term pain, despite previous attempts to forecast the future severity of pain in those already affected^6–8^.

Epidemiological studies have provided valuable insights into the relationship between sociodemographic factors and pain^9,10^, and there is a high overlap between long-term pain and psychiatric conditions, such as depression^11–13^. Typically, older age and female sex are associated with an increased risk of developing long-term pain^14^. Yet, more detailed prospective information about the development of long-term pain is scarce. A large community-based survey conducted in Norway highlighted lifestyle and psychosocial factors, such as obesity, sleeping problems and anxiety, as potential risk factors^15^. Given the limited resources in health care and the large population of individuals with pain, it is vital, for both successful prevention and intervention, to find early determinants of long-term pain.

Theories relating to long-term pain include the bio-psycho-social model^16–18^ where the pain condition is seen as the result from a dynamic interaction between biological, psychological, and sociocultural variables. Recent advancements in health sciences advocate network approaches^19–22^ where this approach allows us to explore the dynamic interplay of biological, psychological, and social factors in chronic illness. Network approaches have recently been applied to study fibromyalgia^23,24^ and depressive and sleep-related nodes in symptom networks have been related to pain severity^25^. Here, we performed a network-based analysis that allowed inclusion of a wide array of baseline factors to determine risk factors for future development of long-term pain. Network analysis was particularly suitable for this study because it allowed us to examine complex interrelationships among a large number of variables simultaneously. Unlike traditional statistical methods that might assess variables in isolation, network analysis provides a holistic view of how biological, psychological, and social factors interact within a cohort and differ between populations. Additionally, networks enhance the interpretability of complex systems by providing intuitive visual representations. In sum, this perspective enables an exploration of how interrelated variables in the bio-psycho-social model of long-term pain differ between groups and interact across levels in those experiencing long-term pain.

In 1969 and 1970, 49,132 late adolescent men (18–19 year olds) were rigorously assessed for physical, psychological and social variables as they underwent military conscription in Sweden^26^, and then followed up in high-quality national registries to sample their health data from adolescence until today relating to whether they have received a long-term pain diagnosis. We selected diagnosis codes where pain is likely the primary cause of disability or ill-health. For example, migraine, low back pain, and osteoarthritis of the knee are all diagnoses where the main symptom is pain (see Table 1). We excluded conditions like cancer, MS, or other diagnoses where pain is usually present but not the main symptom (see Methods for more detail). The network analysis is based on cross-sectional baseline data, with a prospective outlook based on subsequent clinical data. Here, in a sample of 32 913 of this dataset, we present the initial results from our network analyses of these individuals, with a unique life-long perspective. All individuals who developed a long-term pain condition were identified (“Pain”) and compared to those who did not (“No Diagnosed Pain”). To start, we begin by looking at both conditions together (“General”).

**Table 1:** An outline of the different diagnoses and age of onset for the Pain cohort used in this study

	Total	Specific diagnoses				
	Long-term pain	Fibromyalgia /Rheumatism	Irritable bowel syndrome	Low back pain/ lumbago	Migraine	Osteoarthritis of the knee
*n* (%)	9459 (28.7)	3400 (35.9)	193 (2.0)	2978 (31.5)	237 (2.5)	2727 (28.8)
Age at diagnosis Mean ± sd	56.2 ± 9.8	59.8 ± 5.6	54.4 ± 8.2	55.5 ± 8.5	55.7 ± 10.1	52.8 ± 13.2
Age at diagnosis <30 years 30–39 years 40–49 years 50–59 years >60 years	299 (3.2) 487 (5.3) 714 (7.6) 3758 (39.7) 4190 (44.3)	0 (0) 21 (0.6) 84 (2.5) 1402 (41.3) 1892 (55.7)	0 (0) 12 (6.2) 33 (17.1) 90 (46.6) 58 (30.1)	4 (0.1) 171 (5.7) 470 (15.8) 1211 (40.7) 1122 (37.7)	11 (4.6) 14 (5.9) 10 (4.2) 100 (42.2) 102 (43.0)	284 (10.4) 280 (10.3) 117 (4.3) 988 (36.2) 1058 (38.8)

Individuals can receive more than one of the diagnoses, consequently the prevalence of the specific diagnoses exceeds 100%. Additionally, these numbers are derived from inpatient care and specialized outpatient care national registries, capturing only more severe cases, which explains why certain diagnoses (e.g., migraine) are represented but not the overall prevalence.

## Results

### The General network of the Swedish national conscription dataset

Network models consist of *nodes* and *edges*. Nodes represent variables. Here, there are 103 nodes collected in the Swedish national conscription dataset. Nodes are connected by edges, which represent their association. For the edge estimation, we use various non-parametric estimations of statistical relationships due to different types of data (e.g. ordinal, binary, continuous; see methods for longer discussion about these methodological choices). With network models, multiple different properties can be analysed, including analysing global properties: the entire network structure, node-level properties: a measure per node in the network, and edge-level properties: patterns of the connections between nodes. Network models have diverse applications and interpretations. For example, in psychopathology, edge estimation often accounts for the influence of all other variables to isolate relationships between specific variables (e.g., using partial correlations). As a first step toward modelling this type of data with networks, we opted to explore general association patterns (i.e., regular correlations) to understand how biological, psychological, and social variables are more broadly interrelated for the bio-psycho-social model. Thus, in this study, we contrast each of these properties for networks created for those who developed long-term pain (Pain) and those who did not (No Diagnosed Pain). For more detail about the Pain cohort, see Table 1.

**Fig. 1:**
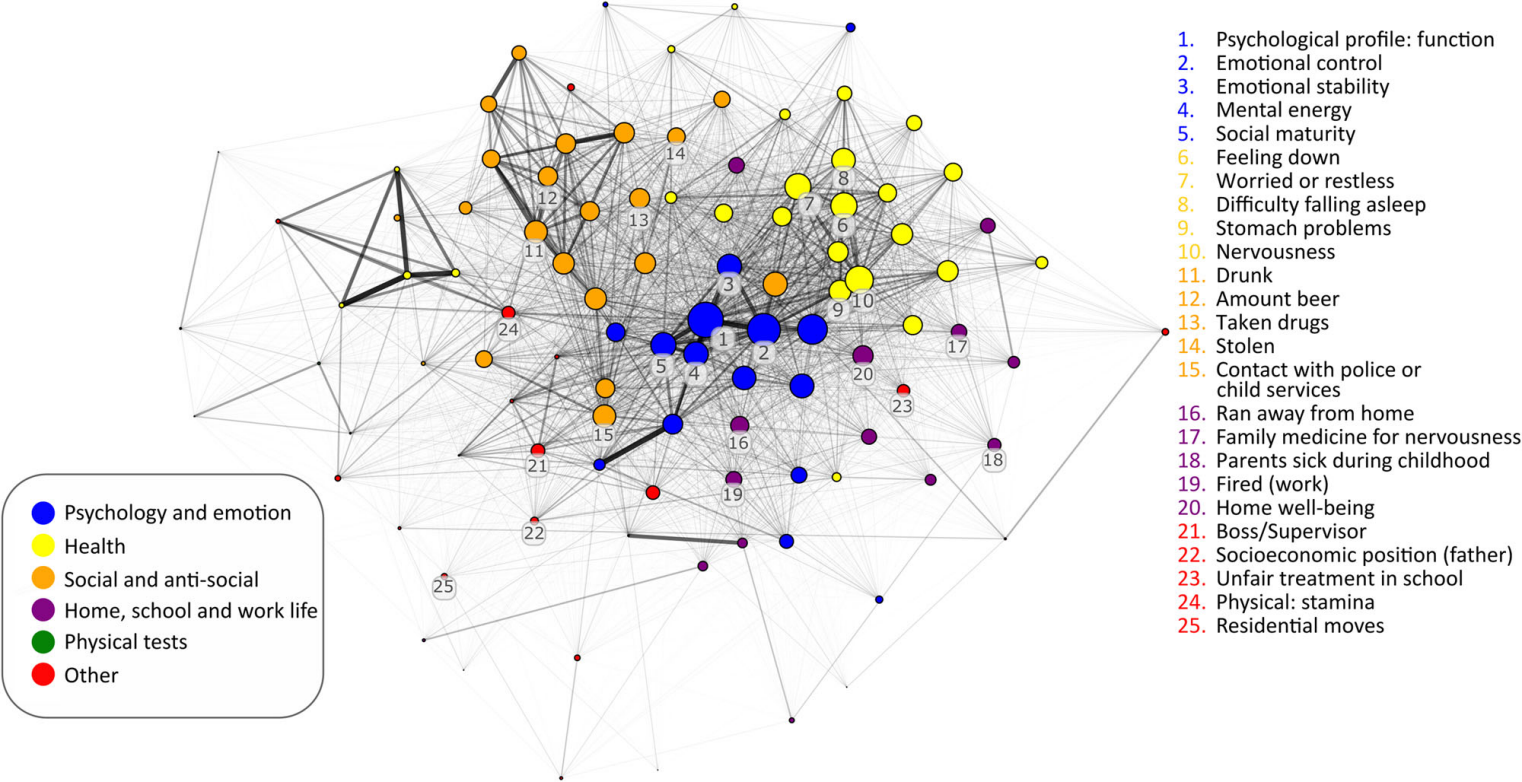
The general network, showing the relation of 103 variables collected during national military conscription for all participants in the cohort. Colours indicate the community (i.e. variables that cluster together). The size of the node shows the strength of a node. Edge width and transparency depict the magnitude of the statistical relationship. Five selected nodes are highlighted in five of the six communities. The sixth community (physical tests, green) all have relatively low strength and are not clearly visible in this static image. An interactive version of the figure is available https://wiheto.github.io/conscription_network/.

In this study, we create networks from 32 913 participants, and 103 different variables at baseline (See Supplementary Table 1 for full list of variables and explanation of variable type). First, we generated a general network that includes all participants (see Fig. 1). This general network illustrates how the variables covary and reveals emerging data communities that cluster into psychological (e.g. *emotional control*), health-related (e.g. *difficulty falling asleep*; this group mainly includes primarily mental health-related variables but also includes *health (current)*, *stomach problems*, and *headache*), social and anti-social (e.g. *amount beer*), school/work-life (e.g. *home well-being*), and physical tests outcomes (e.g. *systolic blood pressure*). The node with the highest strength—meaning it has the strongest overall connections to other variables—was the *Psychological profile: function*, which reflects an evaluation by a psychologist during the conscription interview relating to stress resilience. *Psychological profile: function* was originally assessed to separate individuals deemed suitable for military service from those less suitable for this role.

### Sufficient sample size for comparisons

**Fig. 2:**
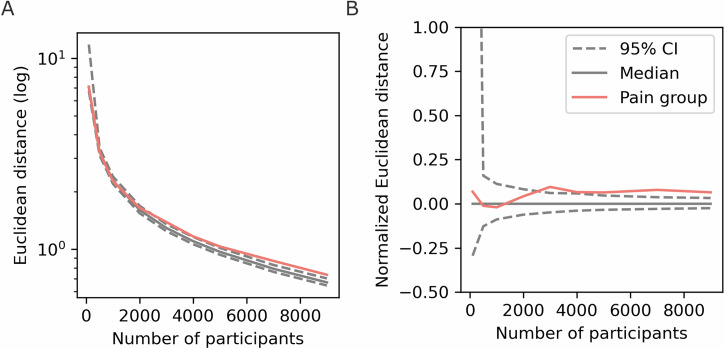
Euclidean distance between the general network, containing all participants, vs the Pain cohorts (red) and randomly sampled networks of similar size (median and 95% confidence interval). **A** The log Euclidean distance, (**B**) The normalised Euclidean difference where the median difference of the bootstrapped distribution was subtracted.

In the present cohort, 9459 (28.7% of the dataset) individuals were diagnosed with long-term pain. We first had to determine whether this is enough individuals to be able to meaningfully compare the Pain and No Diagnosed Pain groups. To do this, we compared the Euclidean distance between the Pain network and the general network. This test is necessary since this is the first time, to our knowledge, that network analyses have been applied to this type of register data, and we expect the Pain cohort to contain some heterogeneous characteristics. The Euclidean distance between networks that only contained Pain individuals (of varying sample sizes) was calculated and compared to the general network in Fig. 1, as well as 1000 permutations of random members of similar sample sizes (Fig. 2). 3000 Pain participants was the threshold when the distance between the networks was different from more than 97.5% when sampling cohorts at random. Hence, the present Pain sample, of 9459 individuals, should be sufficient to find differences despite being a highly heterogeneous population.

Hereafter, the Pain network of 9459 individuals is contrasted to the No Diagnosed Pain network of 23,454 individuals.

### Global measures

Global measures are singular values that we ascribe to the network. First, we tested whether the Pain cohort network differs from the general population network. Here, we tested whether the difference between the Pain network and the entire population network is greater than what would be expected if a network of an identical size was constructed by randomly sampling individuals (as shown in Fig. 2). The difference in Euclidean distance between the network of the long-term pain cohort compared to the entire population network was significant (distance = 0.996, *p* = 0.0012, 95% Bootstrapped CI of null distribution = [0.87, 0.95]). This result entails that the Pain cohort has a network that, as a whole, is significantly different to the General network.

**Fig. 3:**
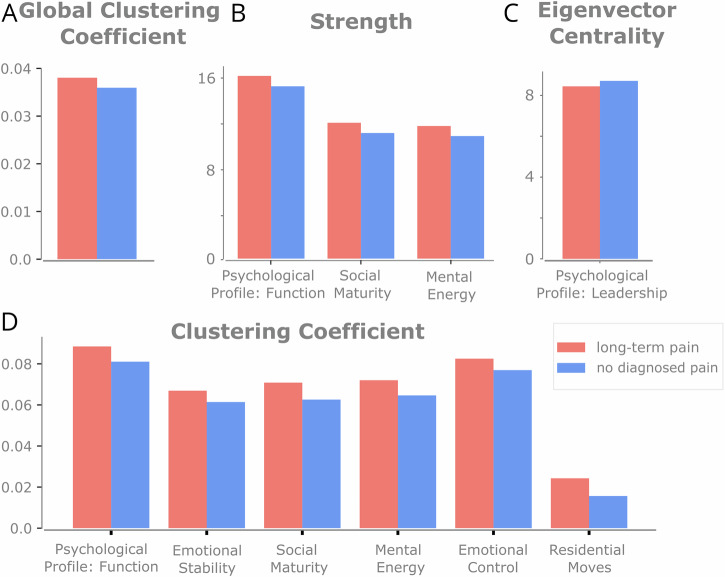
Global and significant nodal network measures comparing long-term pain and no diagnosed pain. Significant results for varying global (**A**) and nodal measures (**B**–**D**) between the Pain and No Diagnosed Pain cohorts. **A** Global clustering coefficient. **B** Strength. **C** Eigenvector centrality. **D** Clustering Coefficient. All nodal properties are FDR corrected. See Supplementary Table 2 for all centrality results.

Next, we wanted to look at the structure of the network of Pain and No Diagnosed Pain cohorts. Here, we contrasted the global clustering coefficient, which is a measure of how much a node’s connections are connected to each other. The global clustering coefficient was significantly higher for the Pain network (Global CC difference = 0.0021, *p* = 0.031, 95% Bootstrapped CI of null distribution = [−0.0020, 0.0019], Fig. 3A), reflecting different topographical properties between the two cohorts—nodes in the network that have strong edges with other nodes in the Pain network are more likely to have stronger edges with each other.

Finally, we tested whether there was any difference in the overall magnitude of edge weights between cohorts. To assess if there were any systematic differences in correlations between the Pain and No Diagnosed Pain networks (e.g. because of sample size), the global network strength was calculated. There was no significant difference between the global strength (Pain: 607, No Diagnosed Pain: 590, *p* = 0.065), which implies that there is no global increase or decrease in edge correlations.

### Nodal properties and differences

Three different outcome measures were used to quantify the properties of network nodes; emphasising slightly different topographical properties: Strength, eigenvector centrality, and clustering coefficient (see Methods). Each of these methods measures the importance of a node in slightly different ways: *Strength* considers the sum of a node’s weights of its edges; *eigenvector centrality* considers a node’s edges and the centrality of connected nodes; The c*lustering coefficient* is a node’s proportion of connections between its neighbours that are also connected.

Regarding strength, 17 of 103 (16.5%) nodes had a two-tailed *p* value below 0.05 (Fig. 3B), which is more than the expected false positive rate (See supplementary table 2). When correcting for the multiple nodes (FDR corrected), three had significant differences where the strength was higher for the pain cohort (Fig. 3B). These nodes all reflect psychological variables.

Next, for eigenvector centrality, only 2 nodes (3.8%) survived the significance threshold of *p* < 0.025 two-tailed (See Supplementary Table 2). After FDR correction, one node was significant (*Psychological Profile: Leadership*); representing larger centrality in the No Diagnosed Pain network (Fig. 3C).

For the clustering coefficient, 24 of 103 nodes (22.3%) were significant at a two-tailed *p* value below 0.05 (See supplementary table 2). A total of 6 nodes had significant differences in their local clustering coefficient (Fig. 3D), as these nodes had more close-knit connections in the Pain network. These variables included psychological (*Psychological Profile: Function*, *Social Maturity*, *Mental Energy*), emotional (*Emotional stability*, *Emotional Control*) and social variables (*Residential Moves*).

Despite the significant differences in network topography, there were no prominent differences in the ranks of centrality for any of the variables. The three nodal measures were correlated between the Pain and No Diagnosed Pain cohorts, and all three approached 1 (r > 0.99 for all three), indicating the results show a magnitude difference in node centrality (e.g. that they increase their overall clustering) but not a reordering of the overall importance of specific nodes.

### Edge-level properties and differences

**Fig. 4:**
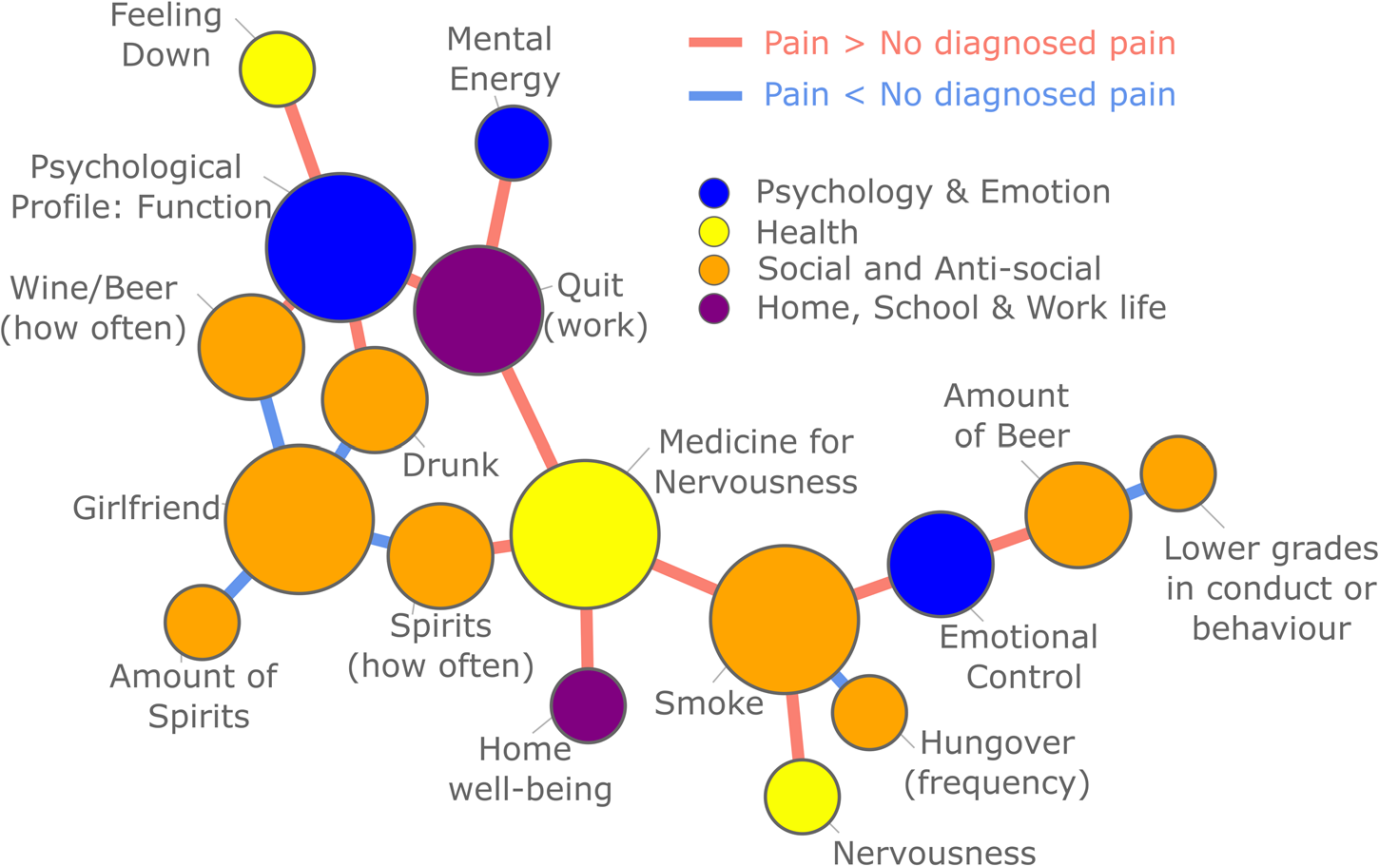
A network visualisation depicts the significant cluster identified after a permutation test comparing edges between the two cohorts. The significant cluster represents a connected component of the network in which all edges significantly differ between cohorts, with the size of this cluster exceeding what would be expected under the null hypothesis of no cluster of connected edges. Red edges indicate greater values in the pain cohort, while blue edges indicate greater values in the No Diagnosed Pain cohort. The threshold for cluster formation was set at 0.04.

For the edge-level, we wanted to test whether there is a group of edges that together form a cluster that is larger than one would expect given the null hypothesis that there is no difference between groups. This is a way of contrasting edges while controlling for the number of statistical tests, as the goal is to find a cluster of edges that differ between cohorts, not specific edges that differ. Testing the sensitivity of the result, we changed the cluster threshold and found that only when the threshold was extremely high (containing the entire network) or very low (containing only a few nodes) no significant cluster was found (See Supplementary Fig. 2). This rejects the null hypothesis, i.e., that there was no significantly large cluster (see Fig. 4, threshold 0.04). To provide more detail about which nodes appear when varying the cluster thresholds, the percent of times an edge was in the Pain network cluster for various thresholds was calculated (Supplementary Fig. 2). This significant cluster entails there are a collection of interconnected edges which together are different between Pain and No Diagnosed Pain cohorts.

The information in Fig. 4 is complex, as the edges display *differences* between the correlations of each cohort. To unpack these values in a little more detail, let us look at one detail of the figure to explain how this should be interpreted. First, let us consider *Smoking* and *Medicine for nervousness*. *Smoking* is a 5-point ordinal scale, where a higher value indicates smoking more often. *Medicine for nervousness* is a binary value, where 1 indicates medicine for nerves has previously been taken. The correlation method used in this instance is point biserial (Pain: 0.136, No Diagnosed Pain: 0.096. Difference: 0.04). Thus, the red edge in Fig. 4 shows a stronger association between smoking and medicine for nerves for those that later developed long-term pain. Similarly, consider *Psychological Profile: Function* (ordinal, where a high-value means low function) and *Drunk* (ordinal, where a high value indicates frequent intoxication). This correlation method is Spearman (Pain 0.107, No Diagnosed Pain 0.065, Difference: 0.042) suggesting that there is a stronger association between alcohol consumption and lower psychological function for the pain cohort. Finally, considering *Drunk* and *Girlfriend* (binary where 1 entails having a girlfriend or engaged), there is a higher correlation for No Diagnosed Pain compared to Pain (Point biserial. Pain: 0.049, No Diagnosed Pain: 0.107, Difference: −0.058). This value suggests that the positive relationship between these two variables is weaker for the Pain group, entailing that those that are frequently intoxicated are less likely to have a girlfriend and vice versa. In sum, these clusters of edges suggest a psychosocial profile of those who are more prone to develop long-term pain.

## Discussion

The present longitudinal study, based on assessments of biological, psychological and social variables in late adolescence, indicates that the risk of developing long-term pain later in life can be indicated already at an early age. Using network-based metrics, our analyses revealed significant differences in the network profiles of adolescent men who later developed long-term pain. The differences were reflected on several network-based outputs, including global, nodal and edge levels, revealing a consistent picture of the pain-associated network profile. This profile demonstrates how those vulnerable to long-term pain have a specific configuration of variables that skew away from the rest of the population, mainly relating to psychosocial aspects. Consistently throughout the analyses, there were psychological (e.g. *Psychological Profile: Function* and *Emotional control* evaluated by a psychologist), and social variables (e.g. alcohol and drug habits) that differed between the groups. The pattern shows that the variables have an increased clustering for the pain cohort. Thus, the intertwining between psychological and social factors appears to deviate for this cohort. it should be noted that the prospective results presented here were based on long-term pain diagnoses recorded in highly reliable national registries throughout the participants’ lives

Almost all significant nodal differences between the cohorts Pain and No Diagnosed Pain represent variables within the community Psychology & Emotion, despite many other types of well-known risk factors included in the study’s 103 variables (e.g., socio-economic background, sleep problems, overall health, violent abuse in childhood^27^). The psychological and emotional variables’ clustering with, and stronger bindings to, social factors (e.g., Drunk and Smoke) in the Pain cohort compared to the No Diagnosed Pain cohort is in line with previous research showing that psychological and emotional difficulties are associated with alcohol^28,29^ and cigarette^28^ consumption. Alcohol and cigarette consumption are also known risk factors for the development of long-term pain^27^. However, such clustering of emotional and psychological variables with social variables has not, to the best of our knowledge, been shown in relation to long-term pain before. Addressing psychological and emotional difficulties (e.g., with cognitive behavioural therapy) may attenuate not only these, but also the associated social risk factors (e.g., Drunk and Smoke) in the symptom network. To target the psychological and emotional risk factors already in adolescence could lead to a shift in the men’s life trajectories and reduce their risk of developing long-term pain.

The theoretical framework in this article rests on the bio-psycho-social model of long-term. Here, have demonstrated that network approaches can elucidate the interaction between psycho-social factors and subsequent incidence of long-term pain. The life course perspective is not well described in studies of long-term pain^30^ as the majority of research studies are cross-sectional and not prospective. Consequently, little is known about the trajectory of pain across the lifespan and how risk factors may differentially act in critical or sensitive periods^31^. In the future, we plan to interrogate our data further by adding assumptions about temporal relationships between specific variables and thereby add a life-course approach to the development of chronic pain. For example, the bio-psycho-social model of chronic pain assumes that a short-term painful event (e.g., knee injury) is associated with differential responses in the psycho-social domain which, in turn, may lead to either spontaneous remission or transition into a long-term pain condition. The dependency of short-term and long-term health events should be included in future analyses in order to increase the attention to the temporal relationship between these variables.

Ultimately, the effect sizes are small. Differences in centrality and weights, while statistically significant, are in a range which should be interpreted appropriately. Considering this data was collected decades before some of these diagnoses, and those who are in the long-term pain cohort can have a multitude of causes from physical impact to chronic pain, we are identifying the *potential risk profile of health determinants* that can be seen at this level. What does this network perspective offer? Both genetic and childhood experiences offer constraints, limiting some outcomes as unlikely while others become more likely. While this approach does not identify necessary causes, it helps identify which types of variables entail proneness *and* the relationship between these proneness variables.

There have been some other recent approaches that have studied health and registry data; one notable recent study is Life2Vec that predicts mortality^32^. Similar predictive approaches are possible with this data in future studies. The approach in this paper differs somewhat as its primary aim is to help identify complex risk categories in an interpretable and interconnected manner rather than prediction.

The study has several limitations. First, the data contains only men. This is because the military service in Sweden only included men in 1969–70. Further, it is well known that long-term pain is more prevalent in women. To that end, we are unaware to what extent these results apply to women. Second, given that we have used the inpatient care and specialized outpatient care register to measure the outcome we only capture the severe cases of pain. Third, some of the social variables may be specific to the time in which they were collected. For example, there was less of a relationship between alcohol and romantic partners for the Pain compared to the No Diagnosed Pain cohorts, which might be indicative of a temporal or cultural phenomenon that might need to be translated to identify equivalent modern behaviour. Finally, this methodology has multiple highly correlated variables which might be better represented as singular latent variables instead. Further methodological considerations are discussed in the methods section. Strengths of the study include the large sample size, the fact that the cohort includes nearly all Swedish men born 1949–1951 (because military conscription was obligatory), the prospectively collected national register data including pain diagnoses in inpatient care from the start of the cohort and outpatient care from 2003. Last, but not least, a major strength of the study is the network analysis approach allowing for calculation and illustration of covarying variables in complex structures. Explicitly, this benefit is evident in several ways: (i) the covariance among various variables is clearly observable, providing insights into their interrelationships; (ii) a comprehensive analysis of global, nodal, and edge-level properties has been conducted, with these distinct levels of analysis converging on similar conclusions; (iii) the complex patterns of variable interactions are rendered interpretable, facilitating a deeper understanding of the data; (iv) the network framework supports predictive modelling, offering insights into potential future outcomes; and (v) the analysis highlights key nodes and edges, identifying the most informative variables and relationships within the population. Future studies can build on this initial work by selecting a smaller, more targeted set of variables and modelling them explicitly to identify specific relationships between variables while controlling for the influence of the remaining dataset.

In this study, we have applied network theory to registry data identifying global, nodal and edge-level differences between the Pain cohort and No Diagnosed Pain cohort. This approach can help identify complex patterns of co-occurring biological, psychological or sociological variables to help identify subgroups and early detection and intervention strategies in the future for long-term pain.

## Methods

### Dataset

The Swedish conscription cohort contains 49,132 male adolescents who underwent compulsory conscription examination for military service during the years 1969–70. Detailed parameters from health examinations, physiological tests, psychological assessment and self-reported questionnaires were used^26^. The cohort is linked to national Swedish registers with prospectively registered diagnoses. The dataset originally consisted of 406 variables. An evaluation reduced this to 103 variables. Variables that were deemed to be unrelated to the aim of the study (e.g., social security numbers or astigmatism on a certain eye), had high rates of missing values, or assessed the same or a very similar construct as other variables, were removed. A list of the 103 variables is available in Supplementary Table 1. Multiple co-authors reviewed the dataset, focusing on variable types and category counts. Some categories were reduced when there were too few participants in extreme categories, or when simplifying the categories made interpretation easier. For example, we reduced ‘Fired (work)’ from three ordinal values (no, yes (once), yes (more than once)) to binary (no, yes) because too few participants fell into the third category, and it was considered easier to interpret the results. This review was completed prior to network construction.

Here we have used 80% of the data. The rest of the data has been locked away from those performing the data analysis, as future work intends to utilise the locked away subset as an independent test dataset for machine learning purposes.

#### Long-term pain cohort

Subjects with long-term pain were those who were in or out patients who had received a diagnosis code according to the Swedish version of the International Classification of Diseases (ICD). Long-term pain was defined using the following ICD-10 codes: G43, G89, K58, M17, M51, M54, M79, and corresponding ICD-8 and ICD-9 codes. These codes represent diagnoses such as fibromyalgia, rheumatism, low back pain/lumbago, migraine and osteoarthritis of the knee.

### Network construction

A considerable number of psychopathological network analyses have used regularisation approaches such as GLASSO. However, non-regularized and non-parametric tests were used for edge estimation. There were several motivations for this approach. First, in a simulation study, non-regularized methods performed better at identifying the underlying network structure when the sample size was large (over 5000)^33^ and regularisation has been shown to have little improvement when subjects greatly outnumber the variables^34^. Second, a recent critique of network analyses in psychopathology argues that the methods should return to simpler metrics with fewer assumptions in as GLASSO fails to control for false positive rates effectively^35^. Third, there are some approaches which aim to combine the mixed-type of data, but these either require ordinal data to be classed as nominal or continuous (e.g. MGM method, which can easily violate assumptions) or are designed for smaller networks. In sum, non-regularized and non-parametric seemed like the appropriate choice for network estimation here.

Another decision to motivate was to choose 103 variables to include in these networks. Above we motivated how we reduced this from 406, but this could have been reduced even more. The reason for choosing 103 variables was as follows: (i) to start with no a priori hypothesis about which variables correlate and allow for a data driven approach which allows us to see which variables cluster (and perhaps represent other latent variables), (ii) wanting to avoid too much of today’s cultural bias about previous generations (iii) some variables where we expect a low correlation or importance (e.g. tests for colour blindness) and we do not expect a large difference between Pain and No Diagnosed Pain cohorts. These effectively act as additional control variables, (iv) Since our edge estimation has no type of regularisation and are pairwise estimates (i.e. no conditional independence), the inclusion of more edges does not reduce the weights of each pairwise edge. This is not problematic as we are not analysing individual edges but differences in edges between groups. The only downside for our analyses here is that adding more variables increases the number of edges and nodes, which increases the number of comparisons. Due to our correction for these number of comparisons, this will more likely inflate our type-II error when correcting for these comparisons. Nevertheless, this type of edge estimation should not be confused with networks made by conditional independence.

With all this considered, the networks were constructed to account for all the various types of data. In general, appropriate pairwise non-parametric measures were chosen to minimise assumptions and as few measures as possible. The data consists of ordinal, continuous and binary variables in the dataset. See Table 2 for the different edge estimation. There are some downsides to this approach (discussed above and in the discussion section).

**Table 2:** pairwise edge estimation method for the different variable types

	Binary	Ordinal	Continuous
Binary	Phi Coefficient	Point Biserial	Point Biserial
Ordinal	Point Biserial	Spearman	Spearman
Continuous	Point Biserial	Spearman	Spearman

#### Multilayer nature of the network

Since there are three different types of edges in this network, this becomes a multilayer network with different edge types. It is important to keep in mind that some of these edges may scale slightly differently. For example, a phi coefficient of 0.25 is typically considered strong while a Spearman of 0.25 may be considered a weak correlation (see ref. ^36^). This variance in edge scaling is not problematic because the tests assessed the difference between cohorts. Additionally, to mitigate any concern that some of this is being driven by the multilayer nature of these different estimates, we also ran the Mixed Graphical Model (MGM) method for network estimation^37^. All default values were used using mgm for R (Extended Bayesian Information Criterion (EBIC) for Lq penalization with gamma set to 0.25). Importantly, this method requires more assumptions and our ordinal values must be classed as nominal values, which was one of our reasons for not selecting this method. Despite the differences in assumptions, variable category, and methodology, there is still a high correlation between the methods. The absolute upper triangle of the adjacency matrices (all subjects) between the method used in this paper and the mgm model correlated highly (Spearman Rho: 0.52, *p* < 0.001) and the centrality of these methods correlate (Spearman Rho: 0.71, *p* < 0.001). Finally, the difference in edge scaling between methods is a motivation for not using any nodal measures based on shortest paths, as any magnitude difference in edges based on edge type makes this measure harder to interpret.

Further, the interactive figure allows filtering by edge type (of the three edges in the multilayer network and the MGM network).

### Sample size for statistical inference

We wanted to determine whether the pain cohort differed from the rest of the population, and also how many people with long-term pain were needed to find a sufficient difference from any other randomly selected group. To achieve this, we varied the sample size from 100, 500, 1000, 3000, 5000, 7000, 9000 people, randomly sampled from the long-term pain cohort. For contrast, 1000 groups of equal size were sampled from the entire dataset. A network was created for each of the groups described above, as well as for the entire dataset. To evaluate the difference, the Euclidean distance of each upper triangle of the connectivity matrix was compared to the network where all subjects were used. Next, global strength (sum of all edges) was calculated to characterise the interconnectedness of the network. Together, these two separate measures gave an indication about how many subjects with long-term pain were needed to find differences greater than randomly created groups and if there was any large difference in edge classification.

### Network analysis

After quantifying the network, three measures to quantify nodal properties were used to understand node-level properties:

*Strength* quantifies the importance of a node by considering the edges to its immediate neighbours. It is calculated using the sum of all the edge weights per node.

*Eigenvector centrality* measures the importance of a node by considering its direct connections and their respective centrality. This is done by identifying the principal eigenvector for the connectivity matrix.

The *clustering coefficient* quantifies the degree to which nodes in a network tend to cluster together. The clustering coefficient of a node is calculated by determining the proportion of connections between its neighbours, relative to the total number of possible connections. The *global clustering coefficient* is the average of all nodes’ clustering coefficient.

All analyses were conducted in python using NetworkX (v 2.8.4^38^) and Brain Connectivity Toolbox^39^ for python (v0.6.0; https://github.com/aestrivex/bctpy).

### Visualisation

The layout of nodes was generated using the Fruchterman Reingold algorithm as implemented in NetworkX. The parameter k was set to 0.03. Louvain community detection was performed on the network to divide the network into clusters of variables that were highly connected with each other. The resolution parameter was set to 1. The community detection algorithm was run 1000 times and the best resulting partition was determined using a consensus matrix with the threshold parameter (Tau) set to 0.5. Fig. 1 is an interactive figure that is available, https://wiheto.github.io/conscription_network/.

### Statistics

Statistical inference employed a non-parametric bootstrapping approach, wherein cohort membership or edges were shuffled (procedures outlined below). The *p*-values were calculated as the proportion of permutations that yielded a test statistic more extreme than the observed value, multiplied by 2 for a two-tailed test. An alpha of 0.05 was used for statistical significance. A 95% confidence interval was calculated using the 2.5th and 97.5th percentiles of the bootstrapped distribution. Values falling outside this range are considered statistically significantCorrection for multiple comparisons in nodal properties was conducted using FDR^40^ with a threshold of q = 0.05. The FDR implementation in multiply (v0.16) was used^41^.

*To assess differences between long-term pain and No Diagnosed Pain cohorts*, 10,000 permutations were generated, maintaining the original cohort sizes. Test distributions were derived by subtracting measures of the first group from those of the second.

*For the network cluster statistics*, a process akin to network based statistics^42^ was undertaken where the goal is to correct for the multiple comparisons by identifying a cluster of edges that differ between groups. Different thresholds were applied to the absolute difference matrices between groups (0.025 to 0.05 in steps of 0.001), calculating the largest remaining cluster size. The null hypothesis posited no distinct cluster of different edges between conditions. Testing involved the largest cluster from 10000 randomly permuted group assignments to examine group differences. This test was one tailed.

*For sample size assessment*, 1000 permutations were executed, randomly assigning group membership while varying group sizes from 100 to 9000. Pain sample membership was randomised to match group sizes.

## Supplementary information

SupplementaryMaterials

## Data Availability

This data is national registry data and cannot be shared openly. The data used are described in Ludvigsson, J. F., Berglind, D., Sundquist, K., Sundström, J., Tynelius, P., Neovius, M. (2022). The Swedish military conscription register: Opportunities for its use in medical research. European Journal of Epidemiology, 37(7), 767–777. 10.1007/s10654-022-00887-0.
