# Supplementary material for: Early signs of long-term pain: prospective network profiles from late adolescence and lifelong follow-up
Source: Npj Ment Health Res. 2025 Feb 13;4:7. doi: 10.1038/s44184-025-00122-0 (PMC11822022; doi:10.1038/s44184-025-00122-0)

Supplementary Table 1

Names	Explanation	Variable_type	Code	Community
Diagnosis	ICD codes used to identify individuals with pain	binary	DIAGNOS	Other
Psychological Profile: Leadership	Psychologists assessed leadership qualities	ordinal	PPRF_BEFL	Psychology & Emotion
Psychological Profile: Function	Psychologists assessed stress resilience	ordinal	PPRF_PF	Psychology & Emotion
Psychological Profile: Intelligence	Psychologists assessed intellligence	ordinal	PPRF_PGRP	Psychology & Emotion
Physical: Height	Height of the conscript	ordinal	KPRF_LNGD	Other
Physical: Stanima	Physical stamina of the conscript	ordinal	KPRF_FYSA	Other
Physical: Sight	Sight quality of the conscript	ordinal	KPRF_SYN	Other
Physical: Sight (colour)	The conscripts ability to see colour	ordinal	KPRF_FARG	Other
Physical: Sight (dark)	The conscripts ability to see in the dark	ordinal	KPRF_MORK	Other
Physical: Hearing	Hearing quality of the conscript	ordinal	KPRF_HORS	Other
Erythrocytes volume fraction	Proportion of blood volume that is occupied by red blood cells (erythrocytes)	continuous	BEVF	Physical tests
Venous blood sedimentation volume	Test of how quickly red blood cells settle at the bottom of a test tube containing a blood sample over a specified period	continuous	VBSR	Health
BMI	Body Mass Index	continuous	BMI	Other
Handgrip	Force exerted when gripping an object with the hand	continuous	HGRP	Health
Knee extension	Bending of the knee extension	continuous	KNST	Health
Elbow flexion	Bending of the elbow join	continuous	ABOJ	Health
Muscle strength	Knee extension strength, handgrip strength and elbow flexion strength	continuous	MFAK	Health
Resting EKG	Resting EKG	nominal	VEKG	Other
Resting Pulse	Resting Pulse	continuous	VPUL	Physical tests
Systolic blood pressure	Systolic blood pressure	continuous	SYST	Physical tests
Diastolic blood pressure	Diastolic blood pressure	continuous	DIAS	Physical tests
End pulse	End pulse	continuous	SPUL	Other
Socioeconomic position (father)	Self-reported information of the fathers occupation: six categories: unskilled workers, skilled workers, low-level non-manual employees,	nominal	SEI_FAR	Other

	intermediate non-manual employees, highlevel non-manual employees, self-employed or farmers, and those not classified			
Emotional stability	Psychologist assessed mental stability and maturity and general ability to handle stress.	ordinal	EMOSTAB	Psychology & Emotion
Social maturity	Psychologist assesed degree of sense of responsibility in group activities, independence, and social extroversion	ordinal	SOCMOG	Psychology & Emotion
Mental energy	Psychologist assessed ability to take initiative, preserve, and be task oriented.	ordinal	PSYKEN	Psychology & Emotion
Emotional control	Psychologist assesed stress tolerance and anxiety, as well as the ability to control nervousness, anxiousness, or aggression	ordinal	EMOKONT	Psychology & Emotion
Enjoy school	Enjoy school	ordinal	SKOLTRI	Psychology & Emotion
Repeated school year	Repeated school year	ordinal	OMKLASS	Psychology & Emotion
Special needs class	If the conscript needed to attend a special needs class	nominal	SPECKL	Psychology & Emotion
Prefect	Been a prefect during school	binary	ORDNMAN	Other
School council	Been in the school council	binary	ELEVRAD	Other
Teacher (agree with)	Get along with teachers	ordinal	LARARE	Psychology & Emotion
Unfair treatment in school	The conscript has felt unfairly treated in school	ordinal	ORATT	Other
Liked by other schoolchildren	Liked by the other school children	ordinal	OMTYCKT	Health
Dislike other schoolchildren	Had children at school that I disliked.	ordinal	EJTYCKT	Health
Truant	Skipped school	ordinal	SKOLK	Social and anti-social
Lower grades in conduct or behaviour	Recieved a lower grade in conduct or behaviour	ordinal	UPFOR	Social and anti-social
Time off for overexhaustion	Taken time off from school or work due to burnout	ordinal	OVERANS	Health
Contact with police or child services	Been in contact with police or child services during childhood.	ordinal	POLISBN	Social and anti-social
Work well-being	Enjoy work	ordinal	ARBTRIV	Home, School and Worklife

Unemployed	Unemployed more than 3 months after finishing school.	nominal	ARBLOS	Psychology & Emotion
Quit (work)	Quite work due to being treated unfairly.	ordinal	ORATTARB	Home, School and Worklife
Fired (work)	Fired from work.	ordinal	SPARKEN	Home, School and Worklife
Agree with supervisor (work)	Getting along with supervisors at work.	ordinal	OVERORD	Home, School and Worklife
Dislike work colleagues	Had colleagues that I did not like.	nominal	ARBKAMR	Home, School and Worklife
Give up hard tasks (work)	Easily give up when working with a difficult task.	ordinal	GERUPP	Health
Like having responsibility	Enjoy having resonsibility for tasks.	ordinal	ANSVAR	Other
Boss/Supervisor	Have been boss/superior over others.	ordinal	BAS	Other
Home well-being	Overall feeling of enjoyment at home.	ordinal	HEMTRIV	Home, School and Worklife
Lived with who?	Mainly been living together with both parents, either parent or someone else.	nominal	VEMBOTT	Home, School and Worklife
Sick during childhood	Frequency of sickness spells during childhood.	ordinal	SJUK	Health
Parents alive	Both parents are still alive.	binary	FORALD	Home, School and Worklife
Divorced parents	Divorced parents.	binary	FORSKIL	Other
Older siblings	Number of older siblings	ordinal	ALDSYS	Other
Younger siblings	Number of younger siblings	ordinal	YNGSYS	Other
Crowded housing	Living in a household with more than two indviduals per room, excluding the kitchen.	binary	TRANGBO	Home, School and Worklife
Parents sick during childhood	Which parents, if any, has been sick during childhood.	nominal	FORSJUK	Home, School and Worklife
Ran away from home	Ran away from home during childhood.	ordinal	RYMT	Home, School and Worklife
Fathers alcohol habbits	Frequency of fathers alcohol consumption.	ordinal	FALK	Social and anti-social
Beaten during childhood	Frequency of corporal punishment during upbringing.	ordinal	STRYK	Home, School and Worklife
Strict upbringing	Measure of how strict ones upbringing was.	ordinal	UPPFOST	
Family's economy	How ones family's finances was percieved.	ordinal	FAMEKON	Health
Girlfriend	Relationship status; either engaged, had a girlfriend or no girlfriend.	nominal	FLICKA	Social and anti-social
Friendships	Measure of the typ of friends; either many friends or a few close friends.	binary	TYPVAN	Health

Spirits (how often)	Frequency of strong spirit consumtion	ordinal	SPRIT	Social and anti-social
Wine/Beer (how often)	Frequency of beer/wine consumtion	ordinal	VIN	Social and anti-social
Had position of responsibility?	Held a position of trust in a sports assocations, scout tropp, political or in another assocation.	binary	FORTROE	Other
Health (current)	Self-reported general health right now.	ordinal	HALSA	Health
Headache	Frequency of headache	ordinal	HV	Health
Difficulty falling asleep	Frequency of difficulty falling asleep	ordinal	SOMNA	Health
Stomach problems	Frequency of stomach problems	ordinal	MAGE	Health
Nervousness	Frequency of feeling nervous	ordinal	NERVOUS	Health
Medicine for nervousness	Frequency of taking medication for nervousness	ordinal	MEDNERV	Health
Family medicine for nervousness	Family member taking medication for nervousness	nominal	FAMNERV	Home, School and Worklife
Feeling down	Frequency of feeling down	ordinal	NERE	Health
Feeling angry	Frequency of feeling angry	ordinal	ARG	Health
Insecurity with others	Frequency of feeling insecure with others	ordinal	OSAKER	Health
Difficulty getting to know people	Frequency of having difficulty getting to know people	ordinal	BEKANT	Health
More sensitive than others	More sensitive than others	ordinal	KANSLIG	Health
Worried or restless	Easily worried or restless	ordinal	OROLIG	Health
Irritable	Frequency of irritability when things have to go fast	ordinal	SNABBIR	Health
Residential moves	Number of places of residence	ordinal	ANTORT	Other
Residental environment	Type of residential environment (city, village etc)	nominal	UPPVORT	Social and anti-social
Tell parents about spare time	Frequency telling parents about spare time	ordinal	TALAFOR	Psychology & Emotion
Number of close friends	Number of close friends	ordinal	ANTVAN	Health
Speaking with friends	Frequency speaking confidently with friends	ordinal	TALAVAN	Psychology & Emotion
Speak to about problems	Who speak to about problems	binary	VEMTALA	Psychology & Emotion
Smoking	Frequency smoking	ordinal	ROKNING	Social and anti-social
How often beer	Frequency drinking beer	ordinal	OOL	Social and

				anti-social
Amount beer	Usual amount when drinking beer	ordinal	MOL	Social and anti-social
How often wine	Frequency drinking wine	ordinal	OVIN	Social and anti-social
Amount of wine	Usual amount when drinking wine	ordinal	MVIN	Social and anti-social
Amount of spirits	Usual amount when drinking spirits	ordinal	MSPRIT	Social and anti-social
Drunk	Frequency drunk	ordinal	BERUSAD	Social and anti-social
Hung over	Frequency hung over	ordinal	BAKIS	Social and anti-social
Taken pick-me-ups (alcohol)	Taken pick-me-ups y/n	binary	ATERST	Social and anti-social
Arrested for being drunk	Frequency arrested for being drunk	ordinal	FYLLERI	Social and anti-social
Pilfer	Frequency pilfer	ordinal	SNATTAT	Social and anti-social
Sniffed (drugs, glue)	Having sniffed	binary	SNIFFAT	Social and anti-social
Taken drugs	Having taken drugs	binary	NARK	Social and anti-social
Previous diagnosis (psychiatric)	Psychiatric diagnosis at conscription	binary	SJN_PSYK	Psychology & Emotion
Previous diagnosis (musculoskeletal)	Musculoskeletal diagnosis at conscription	binary	SJN_MSD	Other

Supplementary Table 2

Names	Strength Pain	Strength No pain	Strength pvals (uncorr)	Eigenvector Cent. pain	Eigenvector Cent. No pain	Eigenvector Cent. Pvals (uncorr)	Clustering Pain	Clustering Nopain	Clustering pvals (uncorr)
Diagnosis	1.704	1.691	0.683	0.000	0.003	0.149	0.005	0.006	0.073
Psychological Profile: Leadership	8.439	8.706	0.296	0.148	0.163	0.000	0.055	0.055	0.990
Psychological Profile: Function	15.285	14.416	0.000	0.248	0.247	0.812	0.088	0.081	0.000
Psychological Profile: Intelligence	5.131	5.411	0.303	0.097	0.109	0.009	0.033	0.036	0.291
Physical: Height	-1.218	-1.115	0.713	0.041	0.042	0.838	0.020	0.019	0.543
Physical: Stanima	-4.648	-3.828	0.005	0.102	0.094	0.095	0.047	0.041	0.005
Physical: Sight	1.910	2.121	0.466	0.004	0.009	0.209	0.010	0.012	0.025
Physical: Sight (colour)	1.042	1.050	0.884	0.002	0.003	0.684	0.003	0.003	0.366
Physical: Sight (dark)	0.850	0.908	0.851	0.014	0.011	0.510	0.010	0.008	0.341
Physical: Hearing	0.884	0.865	0.657	0.012	0.012	0.875	0.007	0.006	0.728
Erythrocytes volume fraction	1.815	1.634	0.495	0.016	0.012	0.428	0.011	0.010	0.418
Venous blood sedimentation volume	0.940	1.216	0.322	0.010	0.015	0.191	0.006	0.009	0.245
BMI	-0.856	-0.572	0.304	0.028	0.027	0.790	0.012	0.010	0.454
Handgrip	2.868	3.010	0.625	0.036	0.039	0.629	0.014	0.016	0.211
Knee extension	4.207	4.073	0.625	0.063	0.065	0.651	0.026	0.026	0.990
Elbow flexion	2.985	3.003	0.960	0.034	0.037	0.668	0.015	0.017	0.165
Muscle strength	4.055	4.065	0.982	0.054	0.057	0.600	0.021	0.021	0.780
Resting EKG	0.784	1.005	0.261	0.006	0.005	0.756	0.005	0.005	0.812
Resting Pulse	1.927	1.445	0.089	0.024	0.019	0.382	0.010	0.012	0.072

**Systolic blood pressure**	0.259	-0.022	0.331	0.012	0.017	0.324	0.012	0.015	0.217
**Diastolic blood pressure**	0.757	0.758	0.993	0.008	0.006	0.719	0.007	0.008	0.746
**End pulse**	-1.650	-1.584	0.801	0.051	0.054	0.590	0.028	0.026	0.280
**Socioeconomic position (father)**	-1.823	-2.100	0.320	0.053	0.060	0.111	0.024	0.026	0.388
**Emotional stability**	10.720	10.255	0.047	0.174	0.174	0.944	0.067	0.061	0.003
**Social maturity**	11.363	10.508	0.001	0.188	0.187	0.810	0.071	0.063	0.000
**Mental energy**	11.093	10.254	0.001	0.186	0.183	0.377	0.072	0.065	0.000
**Emotional control**	14.147	13.587	0.015	0.226	0.227	0.723	0.083	0.077	0.002
**Enjoy school**	10.177	10.055	0.663	0.150	0.156	0.123	0.066	0.064	0.109
**Repeated school year**	3.702	3.701	0.904	0.039	0.039	0.983	0.026	0.024	0.592
**Special needs class**	6.126	6.408	0.520	0.073	0.078	0.251	0.037	0.037	0.821
**Prefect**	6.050	6.302	0.497	0.061	0.068	0.113	0.035	0.036	0.465
**School council**	0.606	0.478	0.407	0.038	0.042	0.182	0.017	0.016	0.446
**Teacher (agree with)**	10.289	10.161	0.655	0.129	0.132	0.538	0.065	0.057	0.021
**Unfair treatment in school**	6.109	5.686	0.069	0.064	0.059	0.155	0.039	0.031	0.008
**Liked by other schoolchildren**	5.178	5.064	0.695	0.074	0.076	0.668	0.032	0.030	0.291
**Dislike other schoolchildren**	6.787	6.839	0.984	0.080	0.084	0.392	0.044	0.045	0.903
**Truant**	10.381	10.274	0.682	0.132	0.134	0.627	0.064	0.058	0.104
**Lower grades in conduct or behaviour**	8.093	8.388	0.428	0.096	0.101	0.284	0.045	0.045	0.834
**Time off for overexhaustion**	8.203	7.753	0.158	0.108	0.103	0.291	0.057	0.047	0.015
**Contact with**	9.797	9.786	0.887	0.121	0.124	0.600	0.056	0.053	0.190

police or child services									
Work well-being	5.362	5.487	0.692	0.068	0.075	0.110	0.038	0.038	0.875
Unemployed	7.230	7.026	0.507	0.090	0.090	0.951	0.048	0.044	0.154
Quit (work)	7.379	6.518	0.015	0.090	0.080	0.042	0.050	0.043	0.068
Fired (work)	7.554	6.922	0.107	0.093	0.085	0.182	0.050	0.047	0.544
Agree with supervisor (work)	6.617	6.680	0.859	0.085	0.091	0.222	0.045	0.045	0.917
Dislike work colleagues	7.149	6.969	0.520	0.092	0.092	0.887	0.047	0.045	0.272
Give up hard tasks (work)	8.672	8.221	0.155	0.107	0.107	0.964	0.051	0.049	0.543
Like having responsibility	-0.486	-0.576	0.420	0.055	0.060	0.096	0.016	0.018	0.012
Boss/ Supervisor	6.189	6.321	0.674	0.061	0.067	0.137	0.026	0.028	0.321
Home well-being	9.115	8.679	0.160	0.120	0.117	0.391	0.057	0.053	0.103
Lived with who?	4.715	4.701	0.959	0.066	0.066	0.956	0.035	0.035	0.773
Sick during childhood	5.559	5.631	0.920	0.072	0.074	0.731	0.036	0.037	0.670
Parents alive	2.624	2.936	0.381	0.022	0.026	0.389	0.017	0.022	0.430
Divorced parents	0.355	0.458	0.777	0.021	0.020	0.829	0.007	0.007	0.854
Older siblings	2.884	3.194	0.360	0.024	0.029	0.247	0.016	0.018	0.477
Younger siblings	2.253	1.962	0.168	0.013	0.006	0.052	0.012	0.006	0.044
Crowded housing	5.192	5.143	0.794	0.056	0.058	0.772	0.038	0.038	0.852
Parents sick during childhood	6.281	5.906	0.219	0.072	0.071	0.697	0.041	0.038	0.487
Ran away from home	8.327	7.844	0.197	0.103	0.097	0.275	0.061	0.050	0.009
Fathers alcohol habbits	6.091	5.791	0.305	0.073	0.071	0.757	0.037	0.034	0.258
Beaten during childhood	5.101	4.501	0.044	0.056	0.047	0.056	0.031	0.026	0.025
Strict	2.179	1.981	0.504	0.011	0.006	0.344	0.007	0.005	0.457

**upbringing**									
**Family's economy**	4.388	4.374	0.960	0.063	0.068	0.355	0.032	0.031	0.565
**Girlfriend**	3.006	3.543	0.060	0.020	0.028	0.117	0.019	0.022	0.055
**Friendships**	3.853	3.681	0.401	0.037	0.033	0.314	0.020	0.018	0.222
**Spirits (how often)**	9.475	9.152	0.265	0.128	0.128	0.969	0.053	0.051	0.213
**Wine/Beer (how often)**	9.147	8.726	0.122	0.114	0.108	0.308	0.045	0.040	0.029
**Had position of responsibility?**	-0.572	-0.332	0.225	0.060	0.062	0.466	0.018	0.018	0.392
**Health (current)**	8.905	9.326	0.184	0.130	0.137	0.064	0.058	0.057	0.704
**Headache**	8.346	8.339	0.953	0.126	0.130	0.280	0.055	0.054	0.403
**Difficulty falling asleep**	10.454	9.967	0.075	0.151	0.148	0.486	0.063	0.059	0.046
**Stomach problems**	9.904	9.312	0.032	0.147	0.142	0.209	0.061	0.056	0.017
**Nervousness**	12.334	11.661	0.013	0.185	0.180	0.159	0.070	0.064	0.004
**Medicine for nervousness**	9.640	8.839	0.023	0.131	0.125	0.162	0.062	0.058	0.243
**Family medicine for nervousness**	7.295	6.777	0.072	0.089	0.084	0.224	0.050	0.048	0.552
**Feeling down**	11.545	11.007	0.040	0.163	0.158	0.175	0.063	0.058	0.028
**Feeling angry**	8.341	7.768	0.041	0.119	0.114	0.166	0.053	0.050	0.066
**Insecurity with others**	8.406	7.846	0.048	0.119	0.114	0.326	0.042	0.036	0.016
**Difficulty getting to know people**	5.760	5.374	0.196	0.086	0.085	0.942	0.031	0.029	0.341
**More sensitive than others**	6.687	6.584	0.711	0.097	0.100	0.594	0.035	0.031	0.144
**Worried or restless**	11.448	10.927	0.050	0.170	0.166	0.283	0.068	0.063	0.005
**Irritable**	9.073	8.837	0.403	0.135	0.136	0.874	0.059	0.056	0.066
**Residential moves**	3.887	3.141	0.007	0.030	0.017	0.004	0.024	0.016	0.002

**Residental environment**	2.369	2.506	0.649	0.009	0.008	0.862	0.013	0.013	0.658
**Tell parents about spare time**	8.646	7.969	0.022	0.125	0.122	0.415	0.059	0.053	0.004
**Number of close friends**	2.957	3.243	0.333	0.029	0.035	0.236	0.016	0.018	0.455
**Speaking with friends**	3.028	2.696	0.256	0.036	0.033	0.674	0.018	0.016	0.283
**Speak to about problems**	4.293	4.392	0.837	0.044	0.047	0.585	0.029	0.025	0.237
**Smoking**	9.604	9.291	0.252	0.137	0.137	0.896	0.059	0.055	0.053
**How often beer**	8.711	8.529	0.515	0.107	0.105	0.842	0.040	0.040	0.646
**Amount beer**	8.799	8.353	0.094	0.116	0.112	0.412	0.048	0.045	0.094
**How often wine**	6.775	6.421	0.234	0.076	0.072	0.412	0.031	0.027	0.088
**Amount of wine**	7.358	7.094	0.361	0.083	0.079	0.550	0.034	0.030	0.071
**Amount of spirits**	7.964	7.761	0.451	0.102	0.102	0.990	0.041	0.038	0.254
**Drunk**	10.014	9.661	0.192	0.132	0.130	0.851	0.052	0.050	0.199
**Hung over**	8.428	8.369	0.843	0.114	0.117	0.421	0.051	0.049	0.275
**Taken pick-me-ups (alcohol)**	7.571	7.075	0.181	0.092	0.090	0.574	0.048	0.044	0.223
**Arrested for being drunk**	7.254	7.499	0.602	0.088	0.093	0.347	0.044	0.041	0.486
**Pilfer**	7.731	7.818	0.841	0.087	0.092	0.335	0.045	0.047	0.311
**Sniffed (drugs, glue)**	8.983	9.048	0.925	0.110	0.113	0.567	0.055	0.053	0.573
**Taken drugs**	8.879	8.649	0.434	0.103	0.104	0.962	0.050	0.048	0.664
**Previous diagnosis (psychiatric)**	12.904	12.287	0.066	0.174	0.172	0.498	0.072	0.069	0.395
**Previous diagnosis (musculoskel etal)**	3.525	3.481	0.785	0.036	0.036	0.981	0.022	0.021	0.804

Supplementary Figure 1

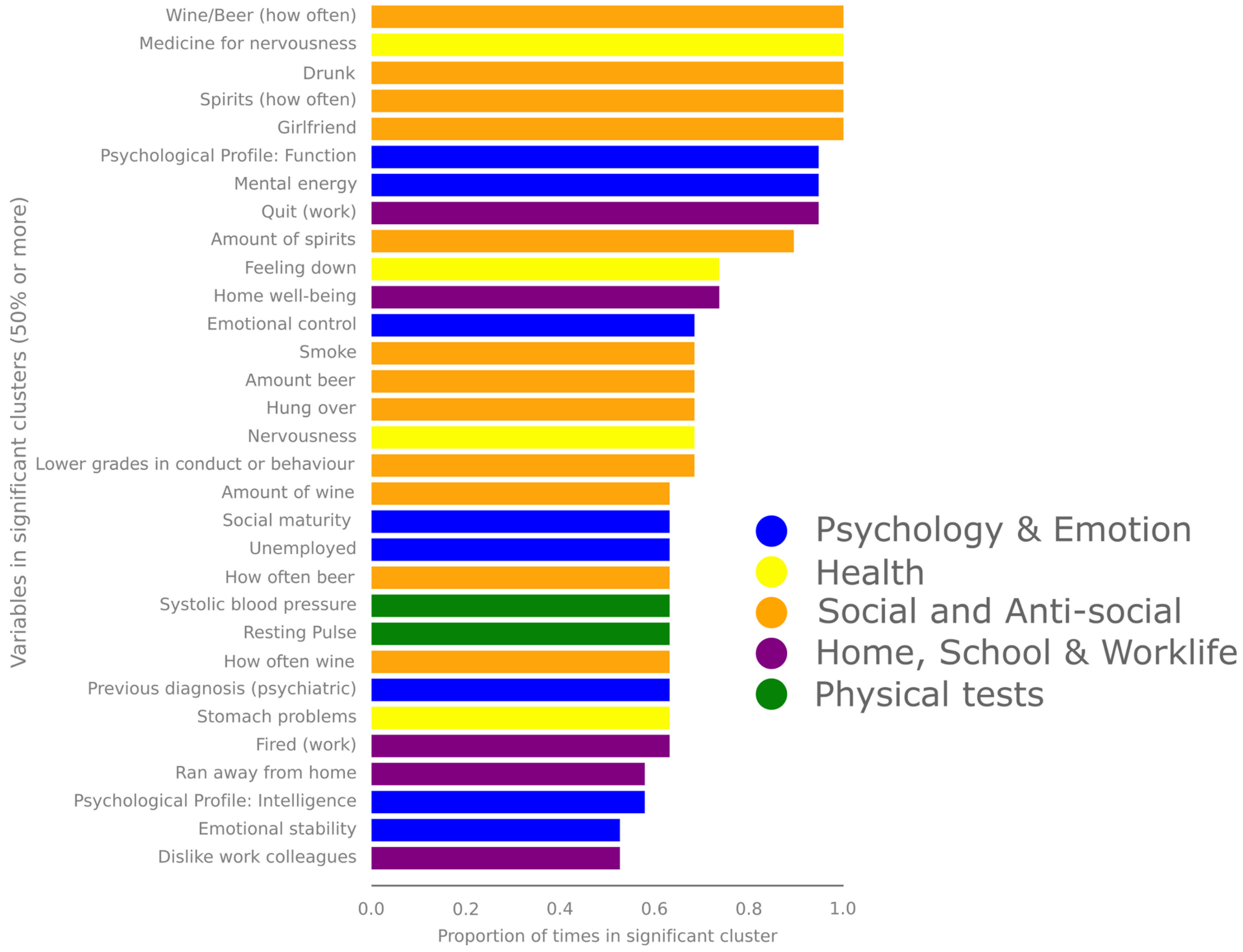

Supplementary Figure 2

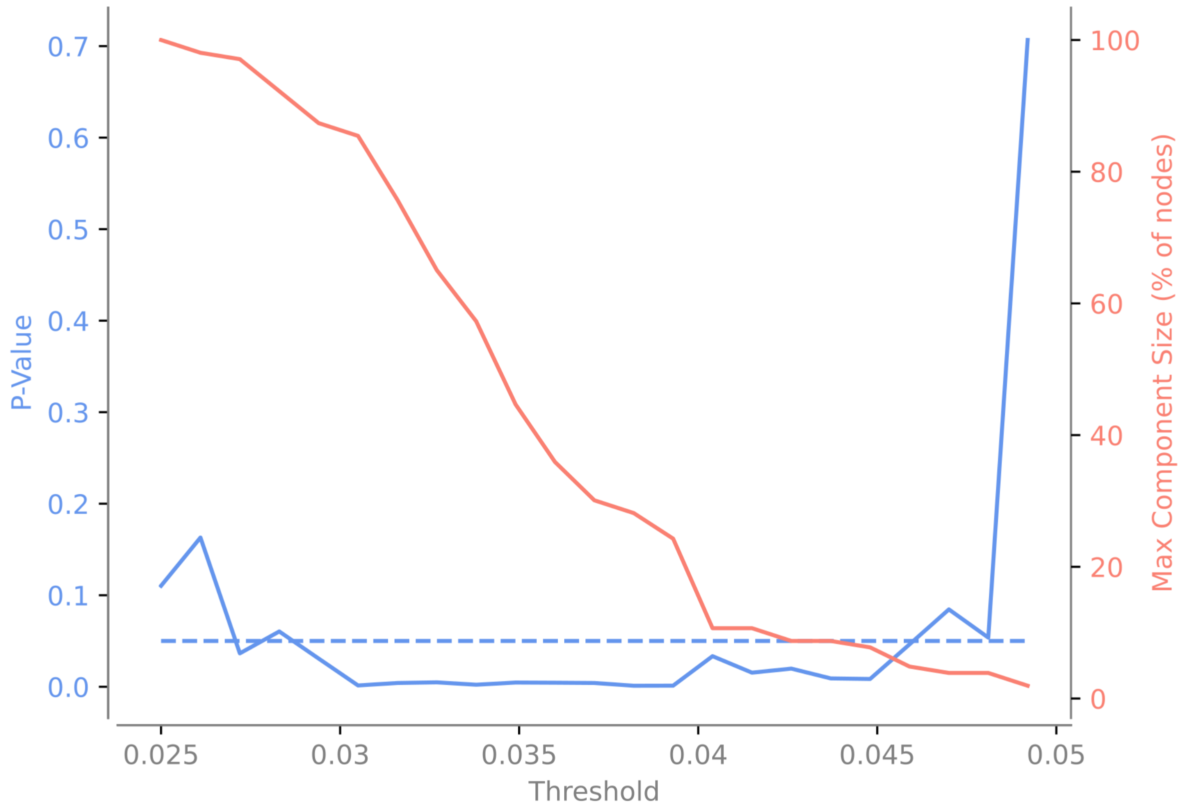

Supplement: Supplementary file 1 — SupplementaryMaterials [file 44184_2025_122_MOESM1_ESM.pdf]
